# SMAD1/5 signaling in osteoclasts regulates bone formation via coupling factors

**DOI:** 10.1371/journal.pone.0203404

**Published:** 2018-09-06

**Authors:** Amy Tasca, Kristina Astleford, Nicholas C. Blixt, Eric D. Jensen, Rajaram Gopalakrishnan, Kim C. Mansky

**Affiliations:** 1 Department of Diagnostic and Biological Sciences, University of Minnesota, Minneapolis, Minnesota, United States of America; 2 Department of Developmental and Surgical Sciences, Division of Orthodontics, University of Minnesota, Minneapolis, Minnesota, United States of America; 3 Departmment of Genetics, Cell Biology and Development, University of Minnesota, Minneapolis, Minnesota, United States of America; Charles P. Darby Children's Research Institute, 173 Ashley Avenue, Charleston, SC 29425, USA, UNITED STATES

## Abstract

Bone remodeling occurs via coupling between bone resorption by osteoclasts and bone formation by osteoblasts. The mechanisms that regulate osteoclast signals to osteoblasts are not well understood. Published studies have reported that BMP signaling in osteoclasts regulate osteoclast coupling targets. To investigate the necessity of canonical BMP signaling on osteoclast differentiation and coupling, we mated *Smad1*^*fl/fl*^; *Smad5*^*fl/fl*^ mice to *c-Fms-Cre* mice. We analyzed male mice at 3 months of age to determine the skeletal phenotype of the *Smad1*^*fl/fl*^; *Smad5*^*fl/fl*^*;c-Fms-Cre* (SMAD1/5 cKO) mice. There was a 1.2-fold decrease in trabecular BV/TV in SMAD1/5 cKO. Analyses of osteoclast serum markers in SMAD1/5 cKO mice, showed a significant increase in CTX-1 (1.5 fold) and TRAP ELISA (3 fold) compared to control mice. In these same mice, there was a 1.3-fold increase in cortical thickness. Consistent with the increase in cortical thickness, we found a 3-fold increase in osteoblast activity as measured by P1NIP ELISA assay from SMAD1/5 cKO mice. To explain the changes in cortical thickness and P1NP activity, we determined conditioned media from SMAD1/5 cKO osteoclast cultures enhanced mineralization of an osteoblast cell line and coupling factors expressed by osteoclasts that regulate osteoblast activity *Wnt1* (4.5-fold increase), *Gja1* (3-fold increase) and *Sphk1* (1.5-fold increase) were all upregulated in osteoclasts from SMAD1/5 cKO compared to control osteoclasts. Lastly osteoclasts treated with dorsomorphin, a chemical inhibitor of SMAD1/5 signaling, demonstrates an increase in *Wnt1* and *Gja1* expression similar to the SMAD1/5 cKO mice. Previous studies demonstrated that TGF-β signaling in osteoclasts leads to increases in WNT1 expression by osteoclasts. Therefore, our data suggest that TGF-β and BMP signaling pathways in osteoclasts could act in an antagonistic fashion to regulate osteoblast activity through WNT1 and other coupling factors.

## Introduction

Pathological bone loss due to an increase in skeletal degradation by osteoclasts relative to bone formation by osteoblasts is the hallmark of diseases such as osteoporosis, periodontitis and cancer-associated bone disease. The development of improved therapies for prevention of pathological bone loss will require a better understanding of the molecular mechanisms that control osteoclast differentiation and activity. Bone morphogenetic proteins (BMPs) are key regulators of bone physiology. Our lab along with others have demonstrated that BMPs are crucial enhancers of osteoclast differentiation [1–4]. Our work and the work of others indicated that osteoclasts express BMP ligands and that BMPs directly enhance osteoclast formation [2, 3, 5–7]. *In vivo* loss of function and *in vitro* overexpression studies revealed that the BMP antagonist *Twisted gastrulation* (*Twsg1*) acts as an inhibitor of osteoclast differentiation [1, 5]. These works established the important role for BMPs in osteoclast differentiation but did not address their mechanism of action. BMPs transmit signals through SMAD transcription factors (canonical BMP signaling), or various protein kinases (non-canonical BMP signaling) [8]. We confirmed the *in vivo* significance of BMPs in osteoclastogenesis by showing increased bone mass due to reduced osteoclast differentiation in a mouse model with a conditional deletion of type II BMP receptor (BMPRII) that primarily disrupted non-canonical MAP kinase pathways while leaving SMADs intact [2].

Our initial studies suggested that SMADs are activated at a later stage of osteoclast differentiation, around the time of precursor cell fusion [3]. SMAD1/5/8 proteins or R-SMADs play an essential role in BMP signal transduction as they are the immediate downstream molecules of BMP receptors [9]. R-SMADs partner with common SMAD, C-SMAD4. The C-SMAD4 is used by both the BMP and TGF-β signaling pathways [9]. Our previously published work investigated the necessity of R-SMAD1/5 and C-SMAD4 during osteoclast differentiation. We flushed bone marrow macrophages from *Smad1* ^*fl/fl*^*;Smad5* ^*fl/fl*^ and *Smad4*^*fl/fl*^ mice. Bone marrow macrophages from the *Smad1* ^*fl/fl*^*;Smad5* ^*fl/fl*^ and *Smad4*^*fl/fl*^ mice were infected with either a control or CRE expressing adenovirus before stimulation with RANKL [4]. We reported that osteoclasts deficient for either SMAD1/5 or SMAD4 expression were TRAP positive but smaller and less active compared to BMMs infected with control adenovirus [4]. Taken together, our data led to a model in which BMP canonical signaling as characterized is necessary for osteoclast fusion and activity [4].

The goal of the current study is to further investigate the function of SMAD1/5 in osteoclasts. Previously we had demonstrated that osteoclasts express detectable levels of *Smad1* and *Smad5* RNA by qRT-PCR but undetectable levels of *Smad8* [4]. Therefore, we focused on characterizing the *in vivo* phenotype of mice conditionally deleted for *Smad1/5* (*Smad1* ^*fl/fl*^*;Smad5* ^*fl/fl*^) in osteoclasts using a CRE mouse line, *C-Fms-Cre* which targets CRE expression primarily in osteoclasts and macrophages [4]. Surprisingly we measured an enhancement in osteoclast differentiation and function and a small decrease in trabecular bone volume fraction in SMAD1/5 deficient mice. At the same time, we also determined that the SMAD1/5 deficient mice had thicker cortical bone and increased bone formation. Based on the changes we measured in cortical bone, we investigated the role SMAD1/5 in osteoclast and osteoblast coupling. Our data suggests that known coupling factors, WNT1, GJA1 and SPHK1, were all up regulated in SMAD1/5 deficient osteoclasts.

## Material and methods

### Breeding of SMAD1/5 conditional knockout

*Smad1* ^*fl/fl*^*/Smad5* ^*fl*/fl^ mice were obtained from Dr. Stephanie Pangas, Baylor College of Medicine, Houston, TX with permission obtained from Dr. Elizabeth Robertson (Oxford University, United Kingdom) and Dr. An Zwijsen (VIB and Center for Human Genetics, KU Leuven, Belgium) who generated the *Smad1* floxed and *Smad5* floxed mice, respectively in a mixed background of C57BL/6 and 129SV as described in [4, 10, 11]. These mice were crossed with B6.129-*Lyzstm1(cre)Ifo/J* mice (*LysM-Cre)* which expresses CRE recombinase in cells of the myeloid lineage (Jackson Labs [12]; *FVB-Tg(Csfr-icre)1Jwp/J*, which expresses CRE recombinase in macrophages and osteoclasts (Jackson Labs [13] and *Ctsk<tm1(cre)>Ska* generated by Dr. Rachel Davey [14], which expresses CRE recombinase in mature osteoclasts. All mice were maintained in a twelve-hour light-dark cycle with a regular unrestricted diet. Procedures described in this study were not expected to produce discomfort but mice were routinely monitored by veterinary staff to alleviate any detected discomfort. Mice were euthanized by asphyxiation with Co_2_.

### Ethics

The use and care of these mice was reviewed and approved by the University of Minnesota Institutional Animal Care and Use Committee, IACUC protocol number 1505-32588A.

### Micro-computed tomography and bone morphometric analysis

Samples were scanned in air using the XT H 225 micro-CT machine (Nikon Metrology Inc., Brighton, MI, USA) at an isotropic voxel size of 6.7 μm. The scan settings were 90 kV, 90 μA, 720 projections, 2 frames per projection, and an integration time of 708 ms. 3D reconstructions were done using the software CT Pro 3D (Nikon metrology, Inc., Brighton, MI, USA). BMP datasets for each scan were made using VG Studio MAX 2.1 (Volume Graphics GmbH, Heidelberg, Germany). Morphometric analysis was completed with the SkyScan CT-Analyser (CTAn) software (Bruker micro-CT, Belgium) according to Bruker micro-CT’s Method Note 2. Method Note 8 was used to select cortical and trabecular regions of interest in an automated manner. All 3D models were created with the CT-Volume (CTVol) software (Bruker micro-CT, Belgium). All micro-CT images presented are at the same magnification.

### ELISA of bone biomarkers

Serum was harvested at time of euthanasia from animals at 3 months of age and subjected to ELISA as per manufacturer’s protocol. Bone resorption was quantitated with CTX (RatLaps EIA, IDS), bone formation was quantitated with P1NP (Rat/Mouse P1NP EIA kit, IDS), and osteoclast number was quantitated with TRAP (Mouse TRAP ELISA, IDS) ELISAs.

### Histological analysis

Femurs were harvested and placed in Z-fix solution overnight. Femurs were decalcified in 10% EDTA, pH 7.4 for 10–14 days, embedded in paraffin and sectioned. Bones were stained for TRAP per manufacturer’s instructions and counterstained with methyl green. Bone surface was quantified using NIH ImageJ.

### Dynamic histomorphometry

50 mg/kg of tetracycline (Sigma-Aldrich) and 25 mg/kg of calcein (MP Biomedicals) was injected by IP at 7 and 2 days prior to euthanasia. Femurs were embedded in methylmethacrylate (MMA), sectioned and analyzed as previously described [2].

### Harvest and culture of primary osteoclasts

Primary bone marrow macrophages were harvested from the femurs and tibiae of 4-week-old WT and *Smad1*^*fl/fl*^*/Smad5*^*fl/fl*^*; C-fms Cre* cKO mice as previously described [4]. Briefly, femurs and tibiae were dissected and adherent tissue was removed. The ends of the bones were cut and the marrow was flushed from the inner compartments. Red blood cells were lysed from the flushed bone marrow tissue with RBC lysis buffer (150 mM NH_4_Cl, 10 mM KHCO_3_, 0.1 mM EDTA, pH7.4) and the remaining cells were plated and cultured overnight in 100 mm tissue culture dishes (TPP, MidSci) in osteoclast media (phenol red-free alpha-MEM (Gibco) with 5% fetal bovine serum (Hyclone), 25 units/mL penicillin/streptomycin (Invitrogen), 400 mM L-Glutamine (Invitrogen), and supplemented with 1% CMG 14–12 supernatant (culture supernatant containing M-CSF). CMG14-12 cells were obtained from Dr. Sunao Takeshita (Nagoya City University, Nagoya, Japan). The non-adherent cell population, including osteoclast precursor cells, was then separated and re-plated in 12-well plates (TPP, MidSci) at 2x10^6^ cells/cm^2^ in osteoclast media supplemented with 1% CMG 14–12 culture supernatant. Two days later cells were refed with 1% CMG 14–12 culture supernatant and 30 ng/mL RANKL (R&D Systems) to stimulate osteoclast differentiation. Cultures were fed every other day for up to 4 days.

### TRAP stain of osteoclast cultures

Primary osteoclasts were fixed with 4% paraformaldehyde and washed with PBS. The cells were then stained for tartrate resistant acid phosphatase (TRAP) expression with using Naphthol AS-MX phosphate and Fast Violet LB salt according to the protocol described in [4]. Cells were then imaged and photographed with light microscopy and the measurements were analyzed using NIH ImageJ.

### Demineralization assay

Primary osteoclasts were plated on Corning Osteo Assay surface plates at a density of 100,000 cells per well. Cells were allowed to fully differentiate. The media was completely removed on day 5 and 100μL/ well of 10% bleach or TRAP stain was added and allowed to incubate at room temperature for 5 minutes. The bleach solution or TRAP solution was then aspirated and the wells were washed twice with 150μL of dH_2_O. The plate was then allowed to air dry completely at room temperature for 3–5 hours. The wells were observed at 4x magnification for the formation of resorption pits and images were captured with light microscopy. Images were measured and analyzed using NIH ImageJ.

### RNA isolation and real-time PCR

RNA was harvested from cells plated in triplicate using Trizol Reagent (Ambion, Life Technologies) and quantified using UV spectroscopy. cDNA was then prepared from 1 μg RNA using the iScript cDNA Synthesis Kit (Bio-Rad) as per the manufacturer’s protocol. Quantitative real-time PCR was performed in duplicate using the MyiQ Single Color Real-Time PCR Detection System (Bio-Rad). Each 20 μl reaction contained 1 μl cDNA, 10 μl iTaq Universal Sybr Green Supermix and 500 nM forward and reverse primers. The PCR conditions were as follows: 95°C for 3 minutes, and the 40 cycles of 94°C for 15 seconds, 56°C for 30 seconds and 72°C for 30 seconds, followed by melting curve analysis (95°C for 5 sec, 65°C for 5 sec and then 65°C to 95°C with 0.5°C increase every 5 seconds). Experimental genes were normalized to *Hprt* or *Gapdh*. Primers amplified with equal efficiencies. All measurements were performed in triplicate, analyzed using the ΔΔCT method. *c-Fos* (Forward) 5’-CCA AGC GGA GAC AGA TCA ACT T (Reverse) 5’-TCCAGTTTTTCC TTCTCTTTCAGCAGA; *Nfatc1* (Forward) 5’ -TCATCCTGTCCAACACCAAA; (Reverse) 5’ -TCACCCTGGTGTTCTTCCTC; *Cathepsin K* (Forward) 5’-AGGGAAGCAAGCACTGGATA; (Reverse) 5’-GCTGGCTGGAATCACATCTT; *Dc-stamp* (Forward) 5’-GGGCACCAGTAT TTTCCTGA; (Reverse) 5’–TGGCAGGATCCAGTAAAAGG; *Smad1* (Forward) 5’-ATTATTGCCGTGTGTGGCG; (Reverse) 5’- TGCAGACCTCCTTCTGCTTG; *Smad5* (Forward) 5’-TGTTGGGCTGGAAACAAGGT; (Reverse) 5’-GTGACACACTTGCTTGGCTG; *Gja1* (Forward) 5’-CCAAGGAGTTCCACCACTTTG; (Reverse) 5’-CCATGTCTGGGCACCTCTCT; *Wnt1* (Forward) 5’-CGCTTCCTCATGAACCTTCAC; (Reverse) 5’-TGGCGCATCTCAGAGAACAC; *Sphk1* (Forward) 5’-GACTTGTCCTGGTGCTGGT; (Reverse) 5’-CCGCACGTACGTAGAACAGA *Bmp6* (Forward) 5’-GGTTCTTCAGACTACAACGG; (Reverse) 5’-GAAGGAACACTCTCCATCA; *Semaphorin 7a* (Forward) 5’-TGGAACTTGGTGAATGACAG; (Reverse) 5’-GGTAGAGTACACTTCATCTCC; *Sclerostin* (Forward) 5’-CGGTGTGTCAACGACAAGAC; (Reverse) 5’-CGGGTGTACCTCTTGCACTT *Efna2* (Forward) 5’-TTTTCCCTGGGCTTTGAGTTC; (Reverse) 5’-GGGTCGGTCCACGAGGTT; *Efnb1* (Forward) 5’TGGACCCTCATGAGACAATGCTGT; (Reverse) 5’ AGGAGATGCCCAAGAATCCCACAA; *Efnb2* (Forward) 5’-TCTGTGTGGAAGTACTGTTGGGGACTTT; (Reverse) 5’-TGTACCAGCTTCTAGCTCTGGACGTCTT.

### Conditioned media treatment of MC3T3 cells

MC3T3 cells were treated with 50% conditioned media from WT or SMAD1/5 cKO osteoclasts. Conditioned media was collected from osteoclast cultures after 4 days of RANKL treatment. MC3T3 cells were fed with conditioned media, alpha-MEM that does not contain ascorbic acid and 1ug/mL ascorbic acid every 3 days for 11 days. MC3T3 cells were obtained from ATCC and maintained under recommended conditions. On the 11th day the cells were fed with conditioned media, alpha-MEM, ascorbic acid and β-glycerophosphate overnight. Next day von Kossa staining was performed, cells were photographed and mineralization was quantitated using NIH ImageJ.

### Statistical analysis

All experiments were completed in triplicate and performed at least three times. The data shown are representative of the mean ± SD of all experiments. Unpaired student’s t-test or 1-way ANOVA analysis followed by a Tukey’s multiple comparison test were used to compare data; p<0.05 indicates significance. Statistical analysis was performed using Prism 5 software for Mac OSX.

## Results and discussion

### SMAD1/5 cKO mice have enhanced osteoclast differentiation and activity

**Fig 1:**
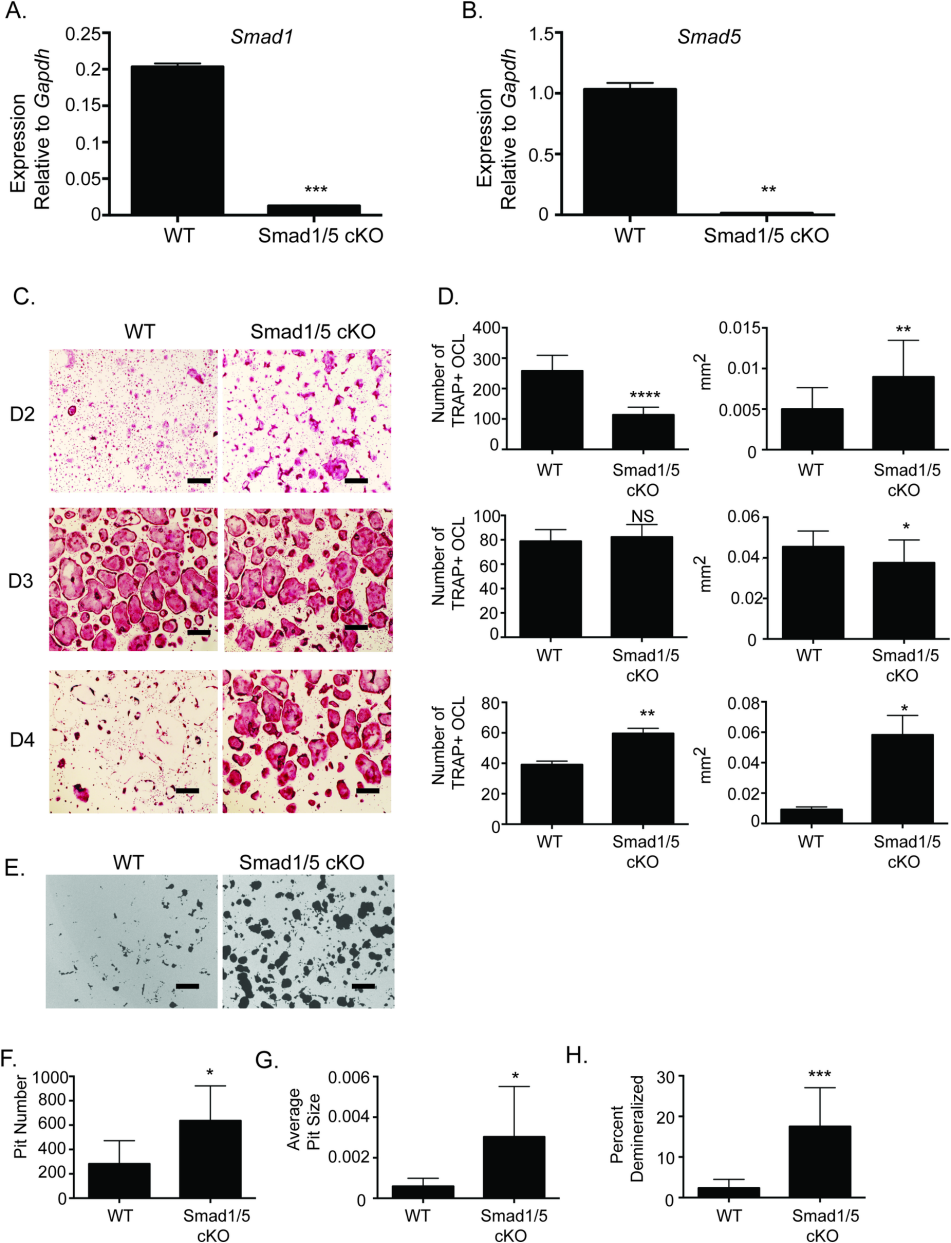
SMAD1/5 cKO mice have enhanced osteoclast differentiation and function. BMMs were flushed from WT or SMAD1/5 cKO mice. BMMs were stimulated with M-CSF and RANKL for indicated days. (A) qRT-PCR was used to measure *Smad1* gene expression following 2 days of RANKL stimulation (B) qRT-PCR was used to measure *Smad5* gene expression following 2 days of RANKL stimulation (C) TRAP stained images of BMMs differentiated with M-CSF and RANKL for 2, 3, or 4 days (D) Quantification of TRAP stained images measuring number and size of TRAP positive osteoclasts. (E) Demineralization activity of WT and SMAD1/5 cKO osteoclast cultures grown on calcium phosphate surfaces. We quantified (F) pit number (G) average site of the pit (H) percent area demineralized. Scale bar 200 μm. Samples were compared using T-test* p<0.05 vs. WT, ** p<0.01 vs. WT,*** p<0.001 vs. WT, **** p<0.0001 vs. WT.

To evaluate osteoclast differentiation and activity of mice that are null for SMAD1/5 expression in the osteoclast lineage, we bred *Smad1*^*fl/fl*^*/Smad5*^*fl/fl*^ mice with *c-Fms-Cre* mice. Culturing osteoclasts from wild type and *Smad1*^*fl/fl*^*/Smad5*^*fl/fl*^*; c-Fms*-*Cre* cKO, herein referred to as WT and Smad1/5 cKO respectively, we confirmed a fifteen-fold decrease in *Smad1* expression (Fig 1A) and fourteen-fold decrease in *Smad5* expression (Fig 1B) in the osteoclasts from the SMAD1/5 cKO mice. We measured osteoclast differentiation and activity of bone marrow macrophages (BMMs) cultured from our mice and then treated with M-CSF and RANKL to stimulate osteoclast differentiation. The resulting TRAP positive multinucleated osteoclasts were measured on day two, three, and four after RANKL stimulation (Fig 1C and 1D). On day two we detected a reduction in the number of osteoclasts in our SMAD1/5 cKO population as they have already started to fuse resulting in larger osteoclasts. On day three the WT population is similar in number compared to SMAD1/5 cKO population; however, there is a slight decrease in the size of the SMAD1/5 cKO osteoclasts. By day four the WT multicellular population is difficult to detect in culture while the SMAD1/5 cKO population still has a significant number of multinucleated osteoclasts. To measure activity of the cultured osteoclasts, BMMs from WT and SMAD1/5 cKO mice were cultured on calcium phosphate coated plates. SMAD1/5 cKO osteoclasts produced significantly more demineralized area compared with WT osteoclasts (Fig 1E). The SMAD1/5 cKO osteoclasts exhibit greater pit number (Fig 1F.), greater pit size (Fig 1G), and greater percent area demineralized (Fig 1H).

### SMAD1/*5* cKO mice have reduced trabecular bone volume

**Fig 2:**
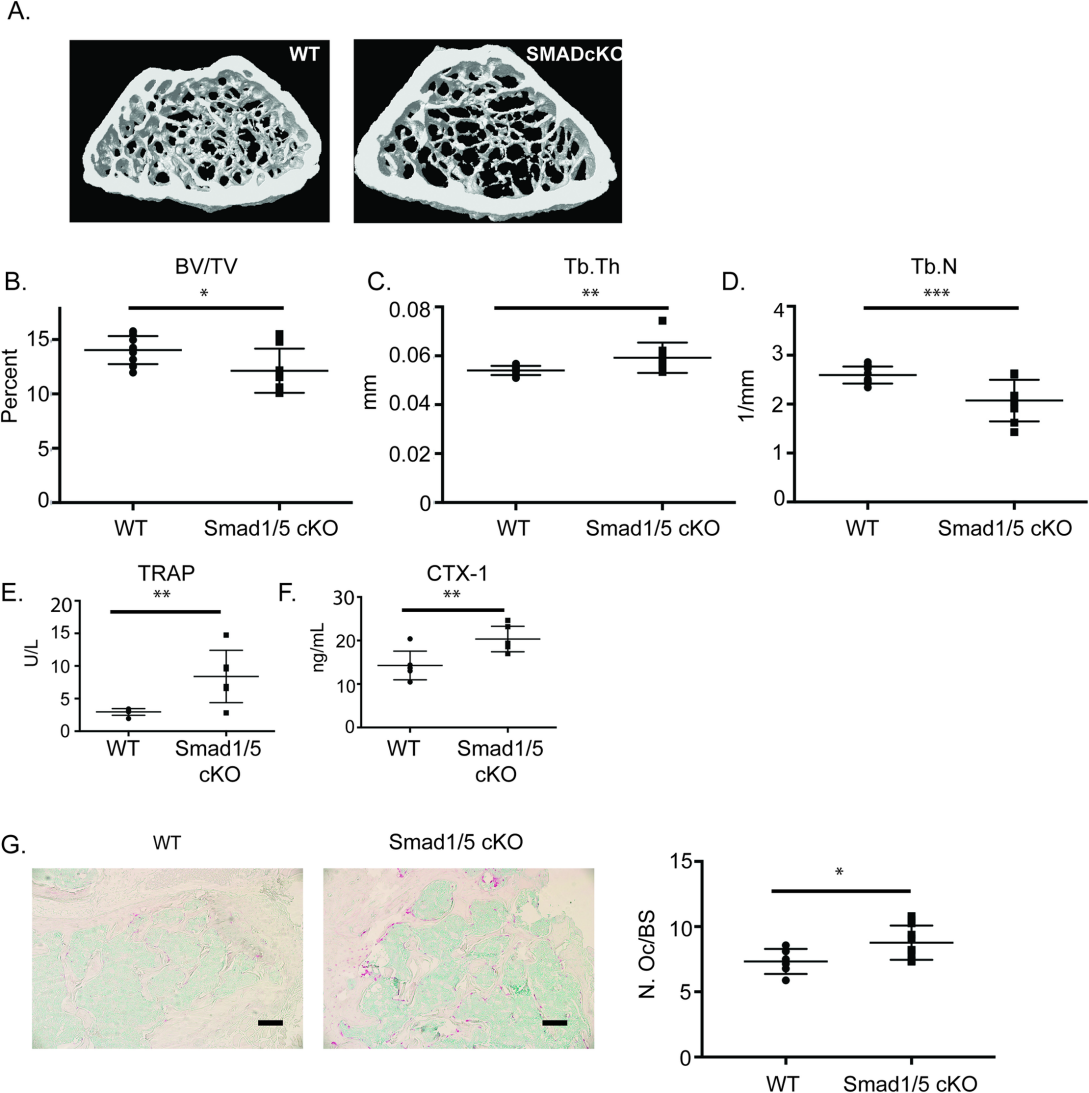
Micro CT analysis of SMAD1/5 cKO mice shows minimal change in trabecular bone. Three-month-old male *Smad1*^*fl/fl*^*/Smad5*^*flfl*^*;Cfms-Cre* and WT mice were analyzed by micro-CT. (A) Representative μCT scans of distal femur from WT and *Smad1*^*fl/fl*^*/Smad5*^*fl/fl*^*;Cfms Cre* mice at 3 months of age. (B) Comparison of bone volume/ total volume (C), trabecular thickness (D) and trabecular number. (E) TRAP ELISA (F) CTX ELISA and (G) histological images and analysis of TRAP stained sections of trabecular bone. Data represent the mean values of 13 WT, 8 KO. Scale bar 200 μm. Samples were compared using T-test * p<0.05 vs. respective WT. ** p<0.01 vs. WT, *** p<0.001 vs. WT.

Since we measured an increase in osteoclast number and activity in *in vitro* cultures from our SMAD1/5 cKO mice, we next analyzed the skeletal phenotype of the SMAD1/5 cKO mice by micro-CT. As shown in Fig 2A, micro-CT analysis of 3-month-old male SMAD1/5 cKO mice revealed a significant decrease in bone volume per total volume (BV/TV, (Fig 2B) compared to WT mice. This decrease in BV/TV was accompanied by an increase in trabecular thickness (Fig 2C) and decrease in trabecular number (Fig 2D) in the SMAD1/5 cKO mice. We did not measure a significant change in any of the trabecular parameters measured in the femurs from the *Smad1*^*fl/fl*^*/Smad5*^*fl/fl*^;*LysM-Cre* mice compared to WT mice (Panels A-D in S1 Fig). Additionally, we analyzed the skeletal phenotype of *Smad1*^*fl/fl*^*/Smad5*^*fl/fl*^*;Ctsk-Cre* mice (Panels E-H in S1 Fig). We measured a trend towards a decrease in BV/TV (Panel F in S1 Fig), and trabecular thickness (Panel G in S1 Fig) between the *Smad1*^*fl/fl*^*/Smad5*^*fl/fl*^*;Ctsk-Cre* compared to WT mice; however, they did not reach significance. These results indicate that the loss of SMAD1/5 expression in osteoclasts affects trabecular bone volume; however, the effect of SMAD1/5 expression on trabecular bone appears to be stage specific in the osteoclasts.

To determine the phenotype of *in vivo* osteoclasts, we measured osteoclast number and resorption in WT and SMAD1/5 cKO animals using serum TRAP (Fig 2E) and CTX ELISAs (Fig 2F). These bone serum biomarkers indicated that *in vivo* osteoclast number and bone resorption in SMAD1/5 cKO animals were increased compared to WT. These finding were consistent with our culture work (Fig 1). Histological sections from WT and SMAD1/5 cKO distal femurs were also analyzed with TRAP staining and we measured a significant increase in number of osteoclasts per bone surface in SMAD1/5 cKO (Fig 2G).

### SMAD1/5 cKO mice have increased cortical bone and bone formation

**Fig 3:**
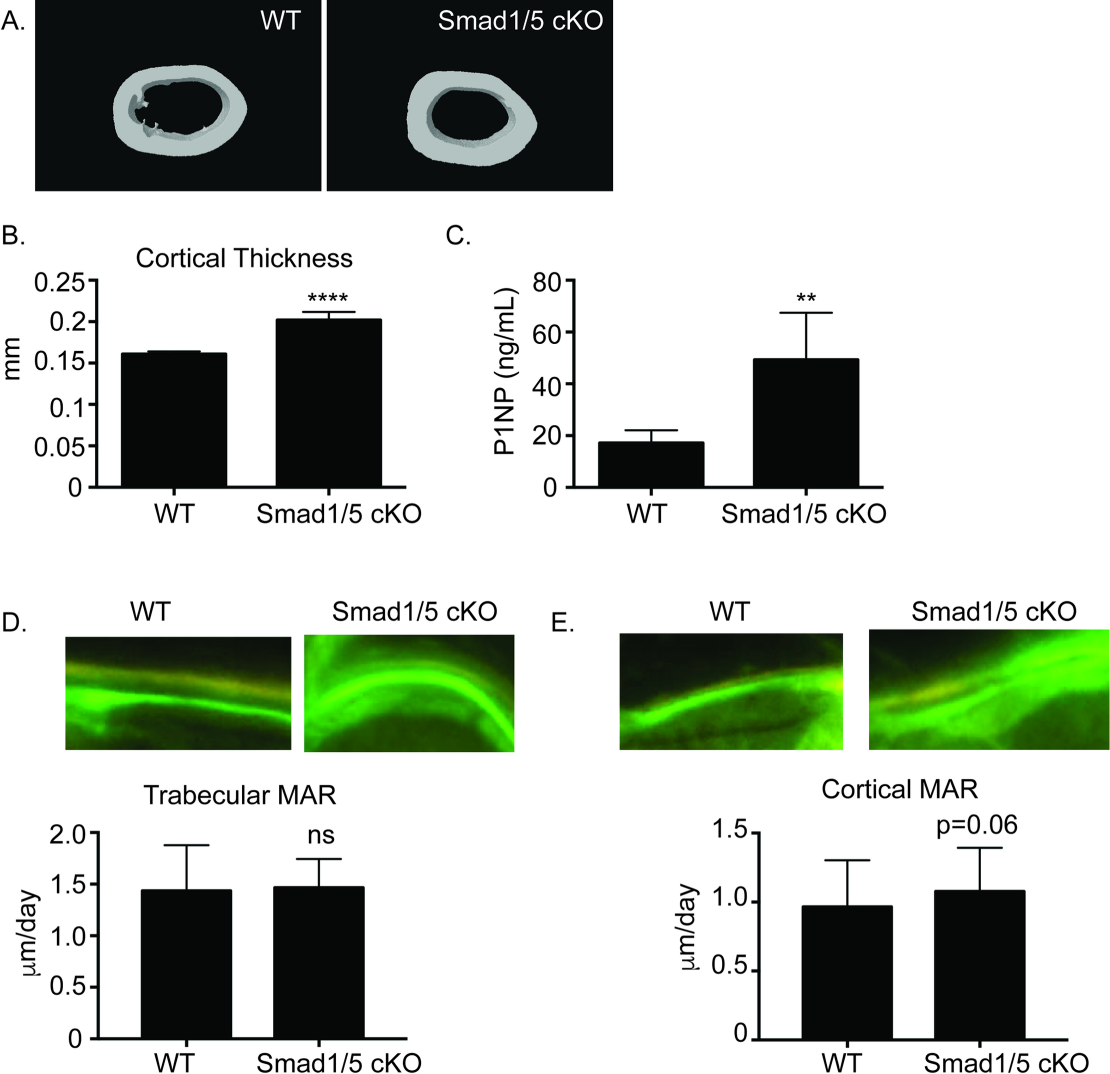
Bone formation is increased in SMAD1/5 cKO mice. Three-month-old *Smad1*^*fl/fl*^/*Smad5*^*fl/fl*^;*c-Fms Cre* male mice were analyzed for cortical bone parameters by micro-CT. (A) Representative μCT scans of cortical bone of femurs from WT and *Smad1*^*fl/fl*^*/Smad5*^*fl/fl*^*;c-Fms Cre* male mice at 3 months of age. (B) Comparison of cortical thickness (C) ELISA analysis of P1NP as a marker of bone formation. Representative images and MAR of tetracycline/calcein labeling from (D) trabecular or (E) cortical bone from WT and SMAD1/5 cKO mice. Samples were compared using T-test ** p<0.01 vs. WT, **** p<0.0001 vs. WT.

Okamoto et al. had previously demonstrated that mice with BMPR1a deleted in mature osteoclasts (*Bmpr1a* ^*fl/fl*^*;Ctsk-Cre* mice) had increased bone formation [15]. Based on these observations, we decided to further analyze the skeletal phenotype of the SMAD1/5 cKO mice by examining the cortical bone. Micro-CT analysis revealed a significant increase in cortical thickness (Fig 3B). To investigate this finding further we looked at bone formation in these mice. ELISA analysis of the serum showed a significant increase P1NP (Fig 3C). P1NP is the N-terminal propeptide of type 1 collagen and a marker of bone formation. To further analyze *in vivo* bone formation, we performed dynamic histomorphometry analysis on trabecular (Fig 3D) and cortical bone (Fig 3E). We measured no significant difference in mineral apposition rate (MAR) on trabecular bone but did measure a trend towards increased MAR on cortical bone in the SMAD1/5 cKO (Fig 3E, p = 0.06).

### Loss of SMAD1/5 increases factors associated with osteoclast activity

**Fig 4:**
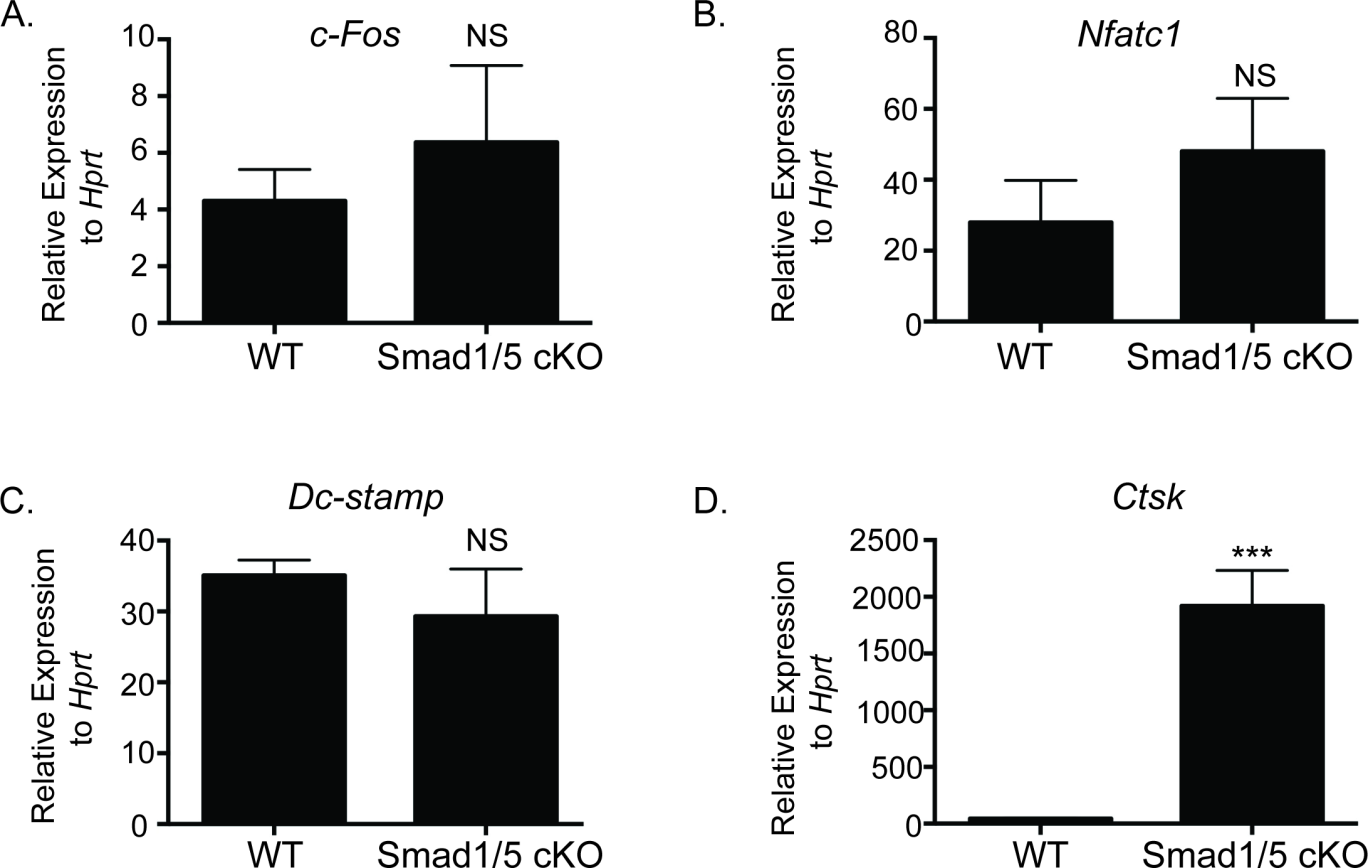
Gene expression of *SMAD1/5* cKO and WT osteoclast cultures. qRT-PCR comparing expression of osteoclast genes from WT and SMAD1/5 cKO mice after 3 days of RANKL treatment. (A) *c-Fos*, (B) *Nfatc1*, *(C) Dcstamp*, and *(D) Ctsk*. Data shown are the mean ± SD of three independent experiments in which gene expression was measured from three wells of each genotype, with each PCR reaction performed in duplicate. Expression of each gene is graphed relative to *Hprt*. Samples were compared using T-test *** p<0.001 vs. WT.

To understand the enhanced bone resorption accompanied with enhanced bone formation in our SMAD1/5 cKO mice, we examined expression of genes important for osteoclast formation and function. We did not note any significant change in *c-Fos* (Fig 4A), *Nfatc1* (Fig 4B), or *Dc-stamp* (Fig 4C). There was a significant increase in *Ctsk* (Fig 4D) expression in BMMs differentiated from SMAD1/5 cKO mice compared to WT. These findings support our data of increased resorption and CTX in the serum of the SMAD1/5 cKO mice.

#### BMP canonical signaling pathway regulates osteoclast-osteoblast coupling

**Fig 5:**
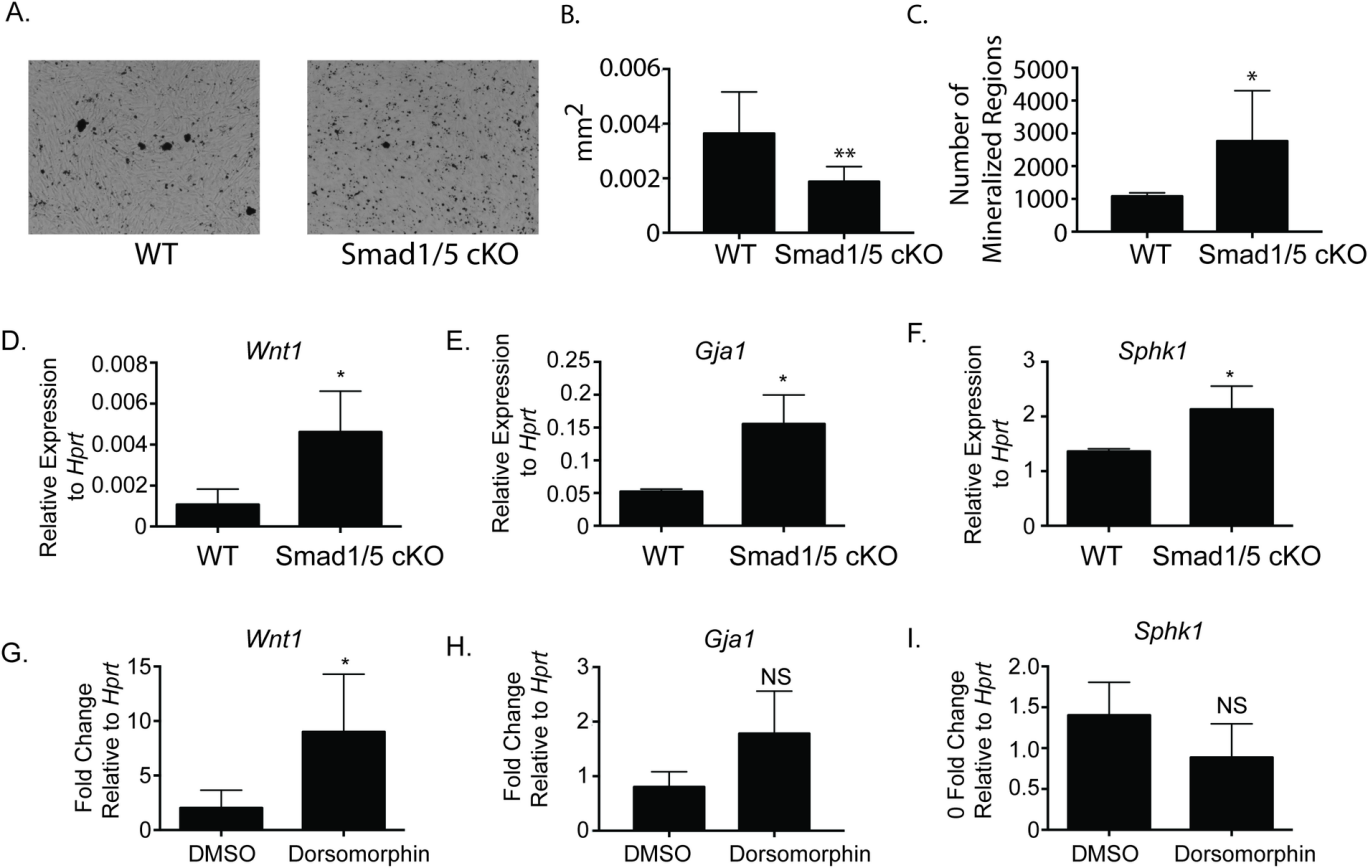
SMAD1/5 signaling negatively regulates osteoclast-osteoblast coupling factors. Conditioned media was collected from WT or SMAD1/5 cKO osteoclasts that had been treated with RANKL for 4 days. MC3T3 cells were treated with conditioned media and ascorbic acid for 12 days. Von Kossa staining was performed on day 12 and quantitated with NIH Image J. (A) Representative images of von Kossa staining of MC3T3 cells treated with osteoclast conditioned media. (B) Size of mineralization nodules (C) Number of mineralization nodules. (D-F) qRT-PCR comparing expression of osteoclast-osteoblast coupling factors from WT and SMAD1/5 cKO mice. (D) *Wnt1*, (E) *Gja1*, and (F) *Sphk1*. (G-I) Mature osteoclasts from WT mice were treated with 1200 nM dorsomorphin or DMSO for 24 hours, and qRT-PCR comparing expression of the coupling factors. (G) *Wnt1*, (H) *Gja1* and (I) *Sphk1*. Data shown are the mean ± SD of three independent experiments in which gene expression was measured from three wells of each genotype, with each PCR reaction performed in duplicate. Expression of each gene is graphed relative to *Hprt* (D-F) and graphed relative to DMSO treatment in (G-I). Samples were compared using T-test * p<0.05 vs. WT, **p<0.001 vs. WT.

Since the CRE mouse lines that we used in our analysis do not affect osteoblast activity directly, we hypothesized alteration in *Smad1;Smad5* expression using *c-Fms-Cre* mouse line affects osteoclast-osteoblast coupling. It has been previously reported that BMP signaling in osteoclasts negatively regulates osteoblast mineralization [16]. To begin to test this hypothesis, we took conditioned media from WT or SMAD1/5 cKO osteoclasts, treated MC3T3 cells with 50% of conditioned media for 12 days and performed von Kossa staining to determine percent of mineralization. We measured an increase in mineralization with the conditioned media from the SMAD1/5 cKO compared to the WT osteoclasts. We see larger areas of mineralization from the WT conditioned media (Fig 5B); however, overall the SMAD 1/5 cKO conditioned media produced more regions of mineralization (Fig 5C). In spite of this result, we cannot exclude the possibility that the difference in viability between the WT and SMAD1/5 cKO osteoclasts (Fig 1) accounts for at least part of the difference in anabolic activity. Next, we examined expression of factors known to be associated with osteoclast-osteoblast coupling. Analyzing RNA from BMMs from our SMAD1/5 cKO and WT mice, we discovered a significant increase in *Wnt1* (Fig 5D), *Gja1* (Fig 5E), and *Sphk1* (Fig 5F) all known factors expressed by osteoclasts that regulate bone formation [16, 17]; however, we did not detect any significant changes in any other known coupling factors such as *ephrins*, *Bmp6*, *semaphorin 7a* and *sclerostin* (S2 Fig). Finally to determine if SMAD1/5 directly regulates expression of *Wnt1*, *Gja1* and *Sphk1*, we treated mature osteoclasts with dorsomorphin, a chemical inhibitor that preferentially blocks SMAD1/5/8 signaling or DMSO for 24 hours [2]. We treated osteoclasts with a concentration of 1200 nM dorsomorphin as previously we had demonstrated that 1200 nM will inhibit SMAD1/5 signaling in osteoclasts [2]. *Wnt1* (Fig 5G) expression in dorsomorphin treated osteoclasts significantly increased six-fold, *Gja1* (Fig 5H) expression increased two-fold; however, *Gja1* increased expression was not significant. Lastly *Sphk1* (Fig 5I) expression decreased one and half fold in dorsomorphin treated osteoclasts; however, it was also not a significant change. This data suggests that the expression of *Wnt1* is regulated by SMAD1/5, and the expression of *Wnt1* is not regulated by the stage of osteoclast differentiation.

Osteoclast and osteoblast formation and function must be carefully regulated to maintain skeletal hemostasis. Our study provides *in vivo* and *in vitro* evidence to support the hypothesis that SMAD1/5 signaling in osteoclasts regulates bone formation via coupling factors. Our *in vitro* analysis of the SMAD1/5 cKO osteoclasts demonstrated enhanced osteoclast differentiation and activity. However, it is interesting that the osteoclasts from the SMAD1/5 cKO mice form multinuclear cells a day earlier in culture but this enhancement by the SMAD1/5 cKO osteoclasts is “overtaken” by the WT osteoclasts after another day in culture. This finding may explain why the SMAD1/5 cKO mice have reduced trabecular bone volume. However, these results were unexpected as our previous *in vitro* study using adenoviral vectors to reduce SMAD1/5 expression demonstrated osteoclast differentiation and activity was reduced in SMAD1/5 Ad-CRE cells [4]. It is not entirely evident the reason(s) why our *in vitro* and *in vivo* model differ in their osteoclast phenotype. One reason might be a timing issue when comparing expression of the CRE expressing adenovirus with the CRE expressing mouse lines. Another explanation might be a change in the bone environment that was not recapitulated in our *in vitro* experiments with the adenovirus expressing CRE.

It has been shown that loss of *Bmpr1a* expression in osteoclasts promotes osteoblast mineralization *in vitro* [16]. We performed a cortical bone analysis and determined that SMAD1/5 cKO mice have increased cortical bone. Bone formation was also increased in the SMAD1/5 cKO mice as measured by P1NP ELISA and dynamic histomorphometry. This led us to investigate how conditional reduction of *Smad1/5* in the macrophage/osteoclast lineage, could significantly alter bone formation. Osteoclasts are able to recruit osteoblasts to sites of bone remodeling through osteoclast-osteoblast coupling [17–20]. There have been numerous potential coupling factors discovered as of recent. Narrowing our search to the regulation of bone formation by osteoclasts involving BMP signaling, we had several potential factors to investigate. GJA1 was described as a downstream target of BMPR1A signaling in osteoclasts that can mediate osteoclast-osteoblast communication during remodeling [16]. We found that GJA1 expression is significantly increased in our SMAD1/5 cKO mice.

Another osteoclast-osteoblast coupling factor that has been characterized is Sphingosine kinase (SPHK1) an enzyme and its reaction product, Sphingosine 1-phosphate (S1P), which are upregulated by RANKL during osteoclast differentiation [19]. S1P has been implicated in osteoclasts’ promotion of bone mineralization [17]. Our SMAD1/5 cKO mice demonstrated a significant increase in SPHK1 expression compared to WT mice. Additionally, SPHK1 has been demonstrated to upregulate RANKL expression on osteoblasts [19]. Some of the changes that we detect in osteoclast differentiation and activity *in vivo* could be explained by changes in SPHK1 expression.

Canonical WNT signaling increases bone formation by promoting osteoblast development [21]. Osteoclasts can stimulate this bone formation by increased activation of the WNT/BMP pathways [17]. Our SMAD1/5 cKO mice exhibit a significant increase in *Wnt1* expression compared to WT mice. Future experiments will need to confirm that WNT1 is one of the secreted factors leading to enhanced osteoblast mineralization as measured in our conditioned media experiments.

**Fig 6:**
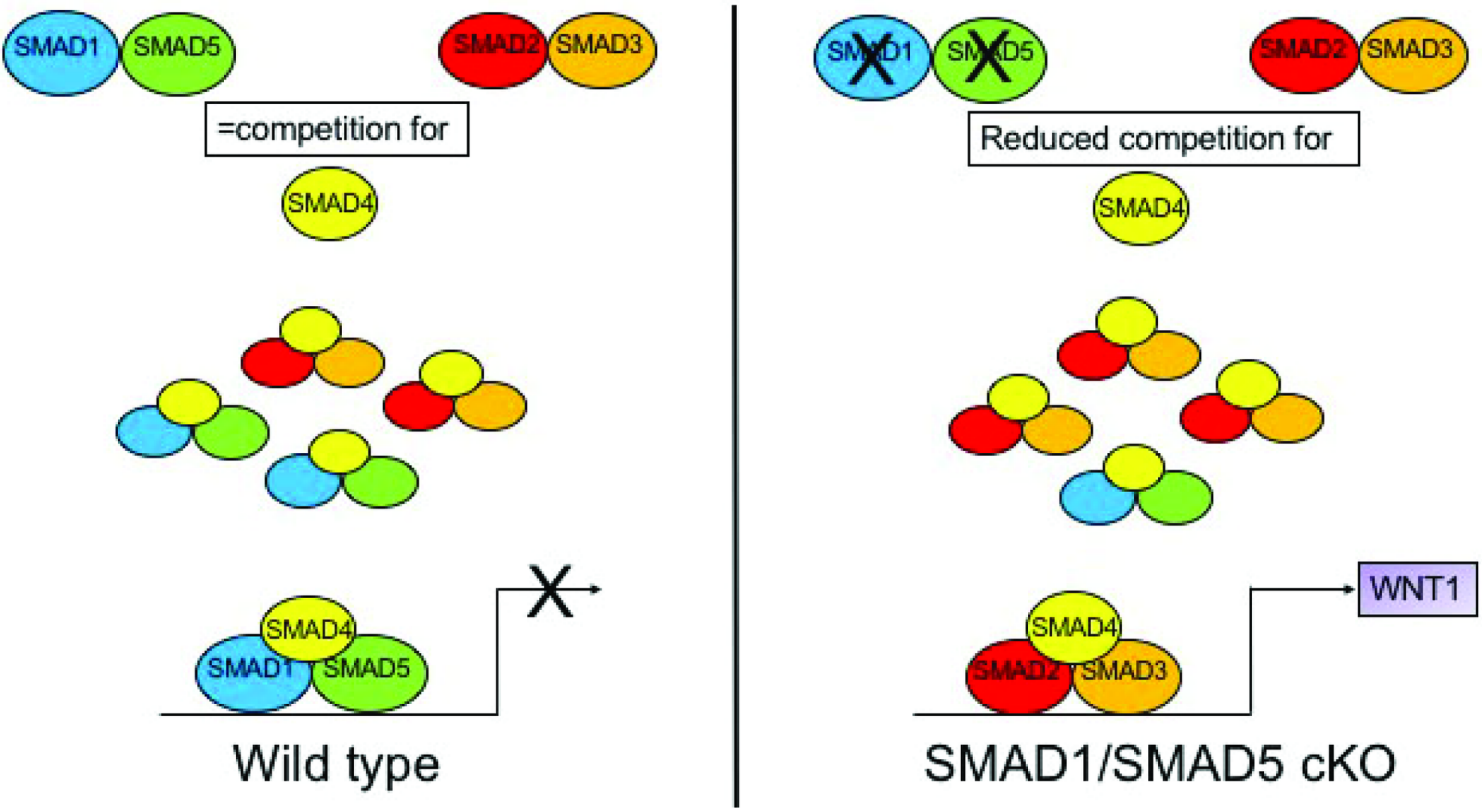
Proposed model of SMAD1/5 regulation of Wnt1a expression in osteoclasts. In WT osteoclasts SMAD2/3 and SMAD1/5 compete for binding of the common SMAD4, C-SMAD4 which limits expression of SMAD2/3 target genes such as *Wnt1*. In SMAD1/5 cKO osteoclasts SMAD2/3 no longer competes with SMAD1/5 for binding to C-SMAD4 and as a result SMAD2/3 target gene such as *Wnt1* are enhanced.

Future studies will be designed to try and understand the mechanism that alters the osteoclast-osteoblast coupling in our SMAD1/5 cKO mice. One potential mechanism is when SMAD1/5 is not present, SMAD2/3 does not have any competition for SMAD4 binding in osteoclasts which enhances TGF-β signaling (Fig 6). Additionally, besides increasing WNT1 expression, the enhanced TGF-β signaling could explain the increased survivability that we observed with our SMAD1/5 cKO osteoclasts (Fig 1, day 4). TGF-β signaling has been shown to promote osteoclast survival through multiple pathways including SMAD2/3 [22]. Weivoda et al. demonstrated that TGF-β signaling in osteoclasts enhances *Wnt1* expression and that mice with a conditional deletion of *Tgfbr2* (*Tgfbr2;Ctsk-Cre* mice) have decreased bone formation as measured by P1NP [18]. The authors hypothesized that lack of TGF-β signaling leads to less *Wnt1* expression by osteoclasts leading to decreased bone formation by osteoblasts [18]. One interesting point on the Weivoda study was that they also measured differences in the skeleton in both male and female mice [18]. In the future, we will analyze the skeletal phenotype of our female SMAD1/5 cKO mice as in the present study, we only analyzed the male skeleton.

## Conclusion

In conclusion, in this study we present *in vitro* and *in vivo* evidence that SMAD1/5 signaling in osteoclasts regulates bone formation via coupling factors. Further study is necessary to provide a clear mechanistic explanation for the role SMAD1/5 plays in regulating coupling between bone resorption and bone formation.

## Supporting information

S1 Fig Skeletal phenotype of three-month-old male *Smad1* ^*fl/fl*^*;Smad5*^*fl/fl*^*;LysM-Cre* or *Smad1*^*fl/fl*^*;Smad5*^*fl/fl*^*;Ctsk-Cre* mice. (A) Representative μCT scans of distal femur from *Smad1*^*fl/fl*^*/Smad5*^*fl/fl*^*;LysM Cre* WT and KO male mice at 3 months of age. (B) Comparison of bone volume/total volume (C), trabecular thickness (D) and trabecular number. Data represents mean values of 9 WT and 10 KO. (E) Representative μCT scans of distal femur from *Smad1*^*fl/fl*^*/Smad5*^*fl/fl*^*;Ctsk Cre* WT and KO male mice at 3 months of age. (F) Comparison of bone volume/total volume (G), trabecular thickness (H) and trabecular number. Data represents mean values of 5 WT and 9 KO.
(TIF)

S2 Fig Expression of Osteoclast-Osteoblast Coupling Factors in SMAD1/5 cKO osteoclasts. qRT-PCR comparing expression of osteoclast-osteoblast coupling factors from WT and SMAD1/5 cKO mice. (A) *Efna2*, (B) *Efnb1*, *(C) Efnb2*, *(D) Bmp6*, *(E) Semaphorin 7A* and *(F) Sclerostin*. Data shown are the mean ± SD of three independent experiments in which gene expression was measured from three wells of each genotype, with each PCR reaction performed in duplicate. Expression of each gene is graphed relative to *Hprt*. Samples were compared using T-test.
(TIF)
