# Supplementary figures and images for: SMAD1/5 signaling in osteoclasts regulates bone formation via coupling factors
Source: PLoS One. 2018 Sep 6;13(9):e0203404. doi: 10.1371/journal.pone.0203404 (PMC6126839; doi:10.1371/journal.pone.0203404)

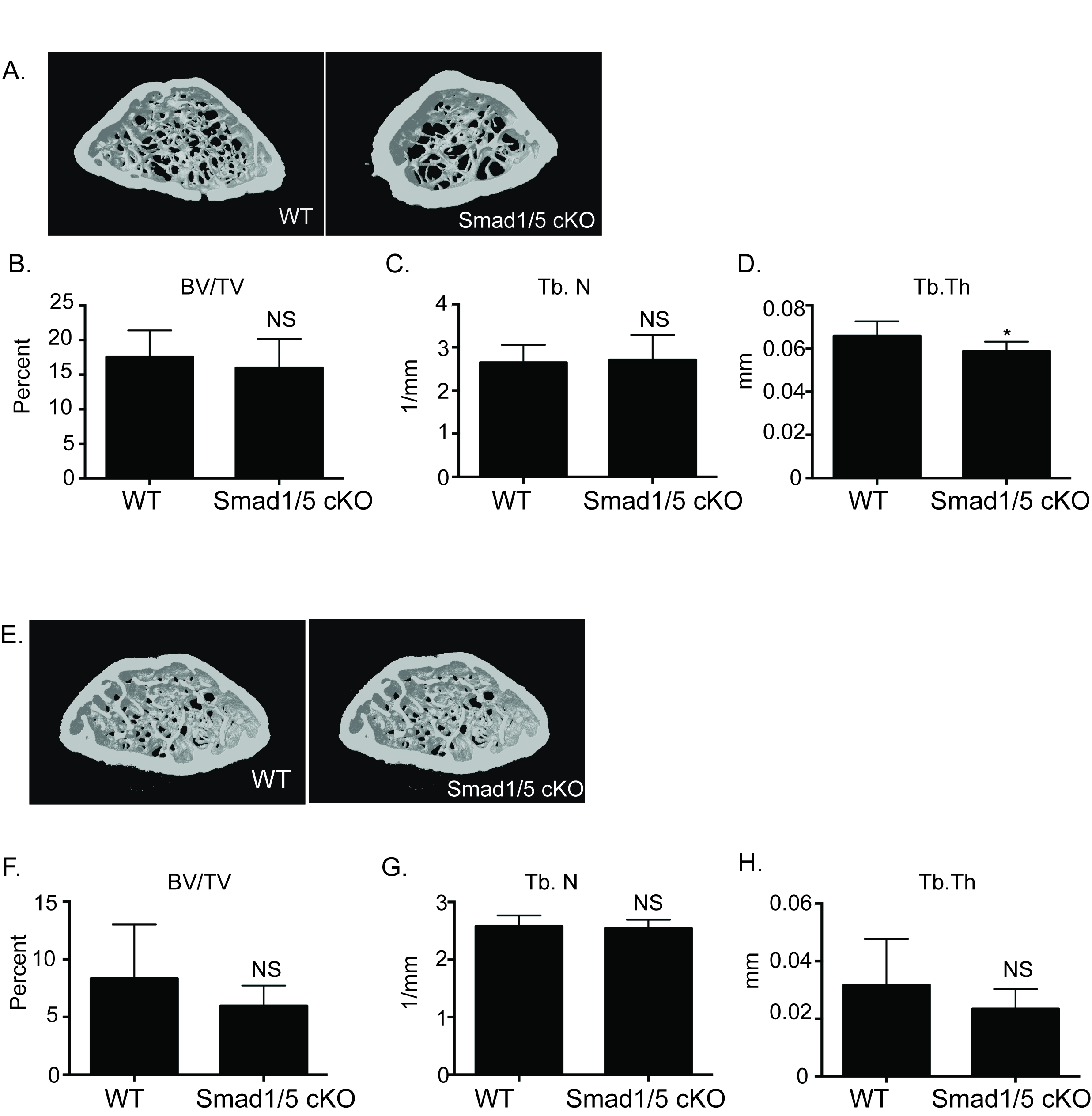

Supplement: S1 Fig — (A) Representative μCT scans of distal femur from Smad1fl/fl/Smad5fl/fl;LysM Cre WT and KO male mice at 3 months of age. (B) Comparison of bone volume/total volume (C), trabecular thickness (D) and trabecular number. Data represents mean values of 9 WT and 10 KO. (E) Representative μCT scans of distal femur from Smad1fl/fl/Smad5fl/fl;Ctsk Cre WT and KO male mice at 3 months of age. (F) Comparison of bone volume/total volume (G), trabecular thickness (H) and trabecular number. Data represents mean values of 5 WT and 9 KO. (TIF) [file pone.0203404.s001.tif]

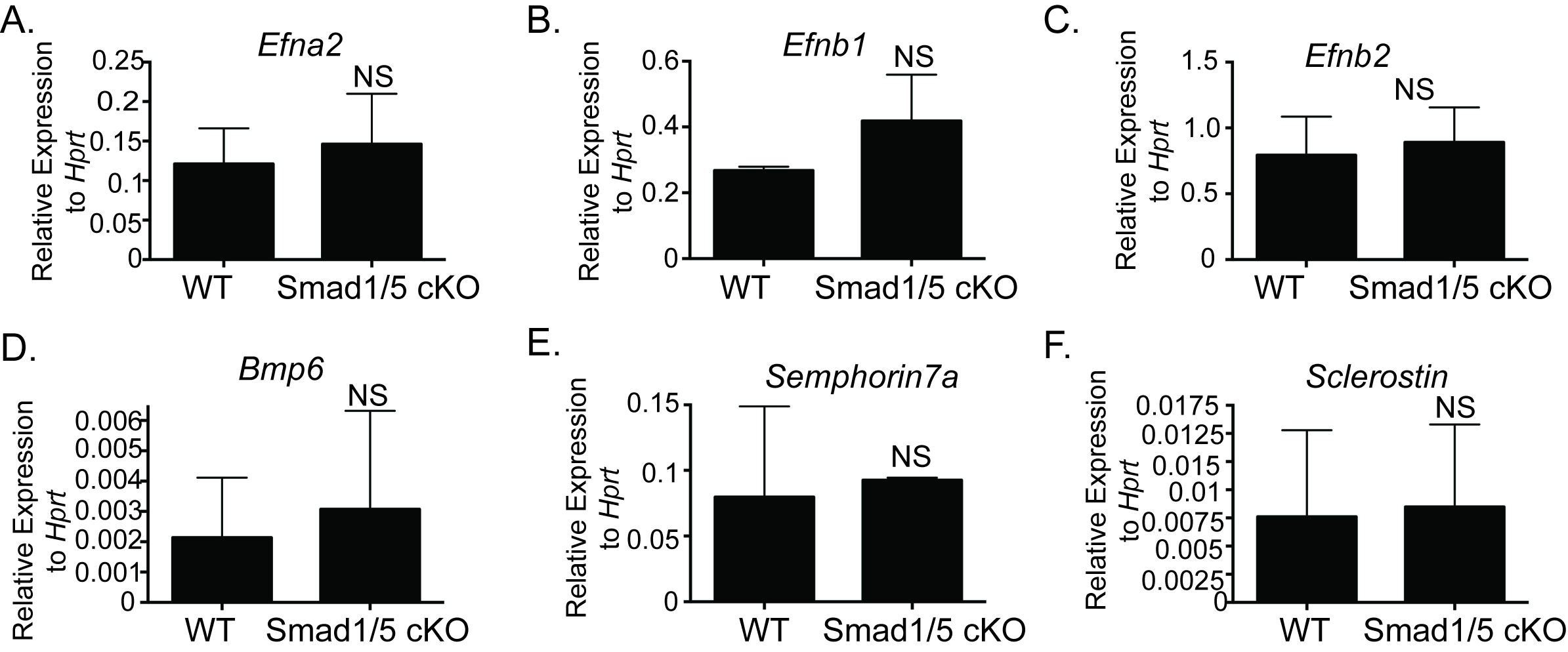

Supplement: S2 Fig — qRT-PCR comparing expression of osteoclast-osteoblast coupling factors from WT and SMAD1/5 cKO mice. (A) Efna2, (B) Efnb1, (C) Efnb2, (D) Bmp6, (E) Semaphorin 7A and (F) Sclerostin. Data shown are the mean ± SD of three independent experiments in which gene expression was measured from three wells of each genotype, with each PCR reaction performed in duplicate. Expression of each gene is graphed relative to Hprt. Samples were compared using T-test. (TIF) [file pone.0203404.s002.tif]
